# Thoracentesis Remarks upon Twenty Cases

**Published:** 1873-05

**Authors:** Austin Flint


### Miscellaneous.

### Thoracentesis.

Remarks from a Report of Twenty Cases. By Prof. AUSTIN FLINT, M. D.

In these twenty cases, thoracentesis was performed *five* times in one case, *three* times in one case, *twice* in eight cases, and *once* in each of the remaining ten cases—the whole number of operations being *thirty-three.*

In fourteen of the twenty cases, the liquid withdrawn from the chest was serous in character; in one case it was sero-sanguinolent, and in five cases it was purulent.

A fatal termination was known to have occurred in nine cases. In one of the fatal cases, the history, after the operation, was imperfect. In seven of the remaining eight cases, causes of death existed aside from the pleuritic affection, as follows: In one case the pleurisy was double, and there existed pericarditis and renal disease. In three cases pneumo-hydrothorax preceded and followed the operation, and in one of these cases the pleurisy was double, and peritonitis was a complcation. Tuberculous disease existed in these three cases. In one case the pleurisy was associated with hydroperitonæum, dependent on cirrhosis of the liver. In one case the autopsy revealed pulmonary apoplexy with cardiac lesions, and in one case the heart was notably atrophied. In the remaining case the affection was empyema. In all the fatal cases the operations produced relief of suffering from dyspnœa, and in one case it apparently averted impending death.

Of the five cases in which the affection was suppurative pleurisy or empyema, in all, the operation was attended with notable relief. In one case it apparently averted impending death. In one case the operately successful as regards recovery, the chest eleven days afterward being free from liquid. In three cases, improvement after the operation was progressive, and recovery was probable at the date of the last record, the patients then passing from under my observation.

In four cases pneumo-hydrothorax existed. In three of these cases the pneumo-hydrothorax was due to perforation incident to tuberculous disease. The pneumo-hydrothorax in two of the three cases was known to have existed prior to the filling of the pleural cavity with liquid, and in the third case this was a rational inference from the previous history. In each of these cases the operation of thoracentesis reproduced the pneumo-hydrothorax, air escaping into the pleura through the perforation of lung. In each of these three cases thoracentesis gave immediate and marked relief from dyspnœa. In one of the cases of pneumo-hydrothorax, this affection was developed after thoracentesis, perforation of lung taking place from without inward. In this case there was complete and permanent recovery.

In four of the cases in which the pleurisy was simple, that is, the effused liquid being serous, the operation was followed by recovery, valvular lesions and enlargement of the heart existing in one of the cases; and in one case the history did not extend beyond a few days after the operation.

Finally, in not one of the twenty cases did the operation fail in giving immediate relief more or less marked, and in not a single instance was there evidence of any ulterior bad effects.

As an addendum to this report, I subjoin, without any discussion, two propositions which seem to me to embody rules of practice as regards the employment of thoracentesis, and also a few remarks on the performance of the operation.

1. Thoracentesis should be resorted to without hesitation or delay whenever an accumulation of liquid or air within the pleural cavity compromises respiration sufficiently to endanger life, or occasion extreme suffering from the want of breath. This rule of practice applies to serous effusion as well as to empyema, and also to cases of pneumo-hydrothorax. Lives are sometimes saved by the operation. Complete recovery may follow ; but the palliation of suffering, under the circumstances stated, furnishes a sufficient indication.

2. Thoracentesis is indicated in cases of pleurisy with considerable serous effusion, although the respiratory function be not compromised sufficiently to occasion any dypsnœa when the patient is at rest, provided the effusion do not diminish speedily under treatment with diuretics, hydragogues and blisters. It is far better to resort to the operation under these circumstances than to persist in the use of the measures just named. These measures are perturbatory, debilitating, and often slow in their operation in the cases in which they prove effectual, whereas the removal of the liquid by puncture of the chest is immediate ; it does not enfeeble the patient, and occasions no constitutional disturbance. Moreover, thoracentesis, resorted to early, has this great advantage: it is likely to be followed by an easy and full expansion of the lung. The long-continued condensation of lung by the pressure of liquid, the investment of the lung by layers of lymph which become dense with age, and adhesions from newly formed tissue, are obstacles in the way of this result.—*Archives of Scientific and Practical Medicine.*

